# Nutritional knowledge, attitude, and practice of professional athletes in an Iranian population (a cross-sectional study)

**DOI:** 10.1186/s13102-023-00776-3

**Published:** 2023-12-04

**Authors:** Amin Hasanpouri, Bita Rahmani, Bahram Jamali Gharakhanlou, Shahabaddin Solaimanian, Saeed Shahsavari, Ahmadreza Rasouli, Saeed Abbasi, Abbas Ebrahimi-Kalan, Tohid Rouzitalab, Zahra Hoseinabadi, Mohammad Reza Shiri-Shahsavar

**Affiliations:** 1 Department of Nutrition, School of Heath, Qazvin University of Medical Sciences, Qazvin, Iran; 2 Student Research Committee, Faculty of Health, Qazvin University of Medical Sciences, Qazvin, Iran; 3 Department of Microbial Biotechnology, Faculty of Basic Sciences and Advanced Technologies in Biology, University of Science and Culture, Tehran, Iran; 4 Department of Basic Sciences, School of Paramedical Sciences, Tabriz University of Medical Sciences, Tabriz, Iran; 5 Department of Pharmacology, School of Medicine, Tehran University of Medical Sciences, Tehran, Iran; 6 Department of Epidemiology and Biostatistics, School of Public Health, Tehran University of Medical Sciences, Tehran, Iran; 7 Nutrition Research Center, Department of Clinical Nutrition, School of Nutrition and Food Sciences, Tabriz University of Medical Sciences, Tabriz, Iran; 8 Department of Neuroscience and Cognition, Faculty of Advanced Medical Sciences, Tabriz University of Medical Sciences, Tabriz, Iran; 9 Department of Biochemistry, Nutrition Therapy, and Community Nutrition, Tabriz University of Medical Sciences, Tabriz, Iran; 10 Metabolic Diseases Research Center, Research Institute for Prevention of Non-Communicable Diseases, Qazvin University of Medical Sciences, Qazvin, Iran

**Keywords:** Nutritional knowledge, Attitude, Practice, Professional athletes, KAP

## Abstract

**Background:**

Proper nutrition is vital in promoting community health, yet insufficient knowledge and improper dietary practices can lead to deficiencies and diseases. Professional athletes depend on optimal nutrition for their performance and recovery, but a lack of understanding can impede their potential. The nutritional status of athletes impacts their overall health and sports performance. Inadequate sports nutrition knowledge may result in suboptimal practices, reducing strength, power, endurance, and immunity. Additionally, disordered attitudes can lead to dietary imbalances and an increased risk of injury. This study, conducted in Qazvin, Iran, examined athletes’ nutritional knowledge, attitudes, and practices. By addressing these factors, there is potential to enhance dietary behaviors and ultimately improve athletes’ performance.

**Methods:**

The study employed a descriptive-analytical, cross-sectional design to investigate the nutritional knowledge, attitude, and practice (KAP) of professional athletes in Qazvin, Iran. A total of 320 purposefully selected professional athletes (68.13% male, 31.87% female) from various sports fields participated in the research. The data collection tool consisted of demographic questions and a KAP questionnaire, including 27 nutritional knowledge questions, 16 nutritional attitude questions, and 14 nutritional performance questions. The quota sampling method was used to estimate sample sizes. Data analysis was carried out by SPSS v.26 using one sample t-test, one-way ANOVA, and Pearson’s correlation coefficient test.

**Results:**

The results showed that nutritional knowledge (the mean value was 79.594 ± 7.015 - the optimal knowledge level = 84) and attitude (the mean value was 23.347 ± 5.300 - the optimal attitude level = 26) for athletes are low, but their nutritional practice (the mean value was 21.788 ± 3.450 - the optimal performance level = 24) was moderate. Also, professional athletes’ nutritional knowledge, attitude, and practice were lower than normal (*p* < 0.05). Significant positive correlations were among knowledge, attitude, and practice (*p* < 0.05). However, there were no significant differences in nutritional knowledge, attitude, and practice among the subjects of different age groups, genders, and sports types (*p* > 0.05).

**Conclusion:**

This study showed that the nutritional knowledge, attitude, and practice of professional Iranian athletes in Qazvin province were low; therefore, the implementation of more theoretical and applied nutrition education, such as using knowledge assessment tools and interventions, irrespective of their age, sport’s field, and gender, is compelling.

**Supplementary Information:**

The online version contains supplementary material available at 10.1186/s13102-023-00776-3.

## Background

Proper nutrition is considered one of the fundamental pillars of community health, as it addresses physical and mental needs, contributing significantly to overall well-being [1]. Unfortunately, inadequate nutritional knowledge and improper dietary practices have emerged as critical factors leading to nutritional deficiencies, malnutrition, and various non-communicable diseases [2]. Professional athletes, in particular, rely heavily on optimal nutrition to support their rigorous training regimens, optimize performance, and promote faster recovery. However, a lack of understanding of proper nutritional principles may hinder athletes from reaching their full potential and achieving peak performance levels.

Several studies have shown that particular age and gender groups exhibit insufficient knowledge about various aspects of nutrition, leading to suboptimal dietary behaviors [3]. Furthermore, the level of education has been recognized as a significant determinant of improved nutritional status, with nutrition education positively correlating with better dietary choices [4, 5]. Nutrition education has proven to be an effective means of preventing malnutrition and promoting better food resource utilization.

Understanding the impact of nutrition on athletic performance is of paramount importance, as it directly influences energy production in response to exercise demands, duration, and intensity [6–8]. Athletes who make poor dietary choices risk compromising their performance potential. Conversely, adopting appropriate diet and nutrition interventions has been shown to positively affect athletic performance [8, 9].

In professional sports, where the margin between success and failure is slim, nutrition assumes a crucial role in determining an athlete’s health, fitness, and overall performance [10, 11]. Proper nutrition facilitates optimal performance, aids in rapid injury recovery, and contributes to the athlete’s ability to reach their full potential [12, 13]. Unfortunately, many athletes, like the general population, may consume sufficient calories but lack essential nutrients, such as vitamins and minerals, leading to suboptimal performance [8, 14, 15].

Athletes’ heightened physical activity demands result in increased energy expenditure and nutrient utilization [16]. They require higher energy intake to fuel their training sessions and support their body’s physiological processes, such as muscle repair and growth [17–19]. Additionally, specific nutrients, such as protein, carbohydrates, fats, vitamins, and minerals, play vital roles in enhancing performance, optimizing recovery, and maintaining overall health in athletes [20]. Therefore, understanding their unique nutritional needs is paramount to ensure they meet the demands of their sport while safeguarding their health.

The interplay between nutritional knowledge, attitudes, and dietary behaviors directly impacts an athlete’s health and performance. Proper nutritional knowledge enables athletes to make informed decisions about their dietary choices, ensuring they meet their energy and nutrient requirements [21, 22]. A positive attitude towards nutrition motivates athletes to consistently adopt and maintain healthy eating habits. On the other hand, poor dietary behaviors, driven by misconceptions or a lack of knowledge, can lead to nutrient deficiencies, compromised immunity, fatigue, and decreased athletic performance [23]. Therefore, fostering accurate knowledge, positive attitudes, and sound nutritional behaviors is crucial for supporting athletes’ overall health and well-being.

A prominent reason for poor nutritional status among athletes is a lack of accurate and adequate nutritional knowledge [24]. Knowledge serves as the foundation for developing desirable attitudes and practices toward nutrition [24]. Additionally, adhering to traditional values and nutritional behaviors can significantly impact the effectiveness of dietary programs for athletes [25, 26]. Accordingly, studying nutritional knowledge, attitude, and practice can play a vital role in choosing the type of training and creating coordination among athletes in implementing nutritional behaviors [23, 27, 28].

Given the importance of nutritional knowledge, attitudes, and practices (KAP) in influencing athletes’ dietary behaviors and performance, it becomes essential to investigate these factors thoroughly. However, current research in this area remains inconclusive, with gaps in understanding nutrition knowledge and adherence among athletes and coaches [29, 30]. There is a need for high-quality research that uses validated tools to measure nutrition knowledge [31]. By evaluating athletes’ nutritional KAP, it is possible to improve the nutritional quality to increase athletic performance [24, 32].

In light of these considerations, this cross-sectional study seeks to assess professional athletes’ nutritional knowledge, attitudes, and practices in Qazvin, Iran. By obtaining essential information about the nutritional KAP of athletes, this research aims to facilitate appropriate planning for targeted nutritional education and interventions. Ultimately, the study endeavors to enhance the nutritional quality among athletes, enabling them to achieve optimal athletic performance, expedited recovery, and ensure their sustained fitness and dietary practices throughout their careers.

## Methods

### Study design

The present research employed a descriptive-analytical study design to assess the nutritional Knowledge, Attitude, and Practice (KAP) of professional athletes in Qazvin, Iran. The study was cross-sectional in nature, allowing for data collection at a specific point in time. This approach enabled a snapshot of the athletes’ nutritional KAP, providing valuable insights into their current understanding and behavior related to nutrition. By utilizing a cross-sectional design, the researchers could investigate the association between nutritional knowledge, attitude, and performance in the athletes without manipulating variables or observing changes over time.

### Study population and sampling method

The study population consisted of all professional athletes residing in Qazvin, Iran. To ensure representative sampling, a stratified random sampling technique was employed. The researchers calculated the quota of athletes in each sports field based on the total number of individuals in that category. Subsequently, participants were selected using the simple random sampling method with the aid of a table of random numbers. This approach aimed to include a diverse representation of athletes from different sports, ensuring that the findings could be generalized to the entire population of professional athletes in Qazvin. In this study, athletes were included if they exercised at least 3 days a week for a minimum of an hour and a half per session, consistently over 3 years, and participated in national or provincial matches, provided they were above 15 years of age. Those with weight fluctuations in the last four months, below 15 years old, or not involved in national or provincial matches were excluded from participation. The inclusion criteria further ensured the homogeneity of the sample by including only athletes who met specific exercise frequency, duration, and competition criteria.

### Sample size

The research scope consists of 1900 people as the initial number used in the calculation. The sample size for the study was determined using Cochran’s sample size determination formula. With a 95% confidence level, a precision level of 0.05, and an estimated proportion of 0.5, the researchers arrived at a sample size of 320 participants. This sample size was deemed sufficient to achieve statistically significant results while considering practical constraints such as time and resources.

### Data collection

Data collection involved the use of two main instruments: initial demographic information and the KAP Questionnaire. Preliminary face-to-face interviews were conducted with the athletes to gather general information about their age, sex, sports field, marital status, education level, career, medical history, supplementation, specific diet, medication, and gastrointestinal tract disorders or diseases (Supplementary file 1 provides the preliminary interview questions used in this study). The KAP Questionnaire, designed by Amirsasan et al. [33]. This questionnaire consisted of three sections. The Knowledge section featured multiple-choice and true/false questions, comprising a total of 27 items. The Attitudes section evaluated participants’ attitudes using a Likert-type scale with a range of 5 points, ranging from “Strongly Disagree” (5) to “Strongly Agree” (1), encompassing 16 questions. The Practices section delved into participants’ nutritional behaviors and practices, employing questions with response options “Yes, I do this” and “No”, along with multiple-choice inquiries to gain insights into their dietary actions. Using a Test-Retest method and the re-administration of the same KAP questionnaire after 4 weeks (test 2) of initial assessment (test 1), the validity and reliability of the questionnaire were confirmed to ensure the accuracy and consistency of the responses provided by the athletes.

### Data analysis

Descriptive statistics were employed to summarize the collected data, with mean values and standard deviations used to present the findings. To ensure the normal distribution of the data, the Kolmogorov-Smirnov test was applied. After confirming data normality, various statistical tests were utilized for comparison purposes. The independent t-test was used to compare means between two groups, the analysis of variance (ANOVA) for comparing means among three or more groups, and the one-sample t-test to compare means with specific values. To explore the relationship between nutritional knowledge, attitude, and performance components, Pearson’s correlation test was employed. This statistical test measures the strength and direction of the linear relationship between two continuous variables. In this study, Pearson’s correlation was used to assess the association between the participants’ nutritional knowledge scores, attitude scores, and performance scores. By calculating correlation coefficients, the researchers were able to determine whether enhancements in knowledge and attitude were linked to improvements in nutritional performance among professional athletes. Prior to analysis, no participants exhibited missing or unreliable data, and consequently, no elimination was necessary to uphold data accuracy and validity.

### Software

For data analysis, the researchers utilized SPSS version 26. The significance level was set at 0.05, indicating that a *p*-value below this threshold would be considered statistically significant. Using SPSS allowed for efficient data processing and rigorous statistical analysis to draw meaningful conclusions from the research findings. Descriptive statistics were used to summarize descriptive data in the form of a mean and a standard deviation.

## Results

### Demographic information

The mean age of the participants was 20.67 years (SD = 2.34), with the majority falling within the age group of 20–25 years and the youngest athletes belonging to the age group of 15–20 years. The participants represented various sports fields, with the highest frequency observed in wrestling (17.81%) and the lowest in badminton (6.25%). The study comprised 68.13% male (*n* = 218) and 31.87% female (*n* = 102) athletes, representing a ratio of 2:1 of male and female genders. Table 1 shows the frequency distribution of participants by age, sport type, and gender groups.

**Table 1:** Frequency distribution of the studied sample by age, sport type, and gender

**Variables**	**Category**	**Frequency**	**Percent**
**Age**	15–20	16	5
	20–25	181	56.56
	Above 25	123	38.44
**Sport**	Football	55	17.19
	Basketball	39	12.19
	Volleyball	47	14.69
	Running	46	14.38
	Wrestling	57	17.81
	Ping-pong	25	7.81
	Badminton	20	6.25
	Swimming	31	9.68
**Gender**	Male	218	68.13
	Female	102	31.87

Most participants were from the wrestling group (17.81%), and the badminton group (6.25%) had the lowest frequency. Highest percentages among the team-based and individual-based disciplines were from the the Football (17.19%) and wrestling group (17.81%), respectively. Table 2 shows the frequency distribution of participants by sport and disciplines.

**Table 2:** Frequency distribution of the studied samples by sport disciplines

**Disciplines**	**Sport**	**Frequency**	**Percentage**
Team-based disciplines	Football	55	17.19
	Basketball	39	12.19
	Volleyball	47	14.69
	Total	141	44.07
Individual-based disciplines	Running	46	14.38
	Wrestling	57	17.81
	Ping-pong	25	7.81
	Badminton	20	6.25
	Swimming	31	9.68
	Total	179	55.92

### Nutritional KAP scores and age, sport, and gender comparison

The results of the ANOVA test in Table 3 to compare the nutritional knowledge, attitude, and performance of athletes in the three age categories of 15–20, 20–25, and above 25 years old indicate that there is no significant difference between the nutritional KAP of different age groups in athletes (*p* > 0.05). The one-way analysis of variance test results in Table 3, comparing the KAP of athletes in various sports, indicates no significant differences among athletes’ nutritional knowledge, attitude, and performance between different sports (*p* > 0.05). The results of the one-way ANOVA in Table 3 to compare the nutritional knowledge, attitude, and performance of athletes in the two gender groups of men and women indicate that there is no significant difference between nutritional KAP of male and female athletes (*p* > 0.05).

**Table 3:** Comparison of nutritional KAP by age, sport type and gender

**Variables**	**Category**	**Number**	**Mean**	**Standard Deviation**	**CI 95%**			Effect size^*^
					**Lower**	**Upper**	***p*-value**	
Knowledge	15–20	16	88.063	8.729	83.411	92.714	0.966	0.0002
	20–25	181	88.481	7.915	87.320	89.641		
	Above 25	123	88.342	5.182	87.417	89.266		
Attitude	15–20	16	29.313	4.990	26.654	31.971	0.832	0.001
	20–25	181	28.536	6.040	27.650	29.422		
	Above 25	123	28.740	4.061	28.015	29.465		
Performance	15–20	16	25.875	2.825	24.369	27.381	0.756	0.002
	20–25	181	26.127	3.593	25.600	26.654		
	Above 25	123	26.382	3.323	25.789	26.975		
Knowledge	Football	55	80.236	12.506	55.724	104.748	0.988	0.004
	Basketball	39	79.717	5.072	69.776	89.658		
	Volleyball	47	84.659	5.100	74.663	94.655		
	Running	46	81.087	5.265	70.768	91.406		
	Wrestling	57	80.631	5.521	69.810	91.452		
	Ping-pong	25	80.160	5.210	69.948	90.372		
	Badminton	20	80.750	5.270	70.421	91.079		
	Swimming	31	83.741	5.409	73.139	94.343		
Attitude	Football	55	24.272	9.135	6.367	42.177	0.672	0.015
	Basketball	39	25.359	3.956	17.605	33.113		
	Volleyball	37	24.425	3.669	17.234	31.616		
	Running	46	25.021	3.997	17.187	32.855		
	Wrestling	57	24.052	4.381	15.465	32.639		
	Ping-pong	25	26.360	4.221	18.087	34.633		
	Badminton	20	24.300	4.835	14.823	33.777		
	Swimming	31	24.193	3.815	16.716	31.670		
Performance	Football	55	22.363	3.423	15.654	29.072	0.859	0.011
	Basketball	39	22.128	3.941	14.404	29.852		
	Volleyball	47	22.148	3.106	16.060	28.236		
	Running	46	22.478	3.716	15.195	29.761		
	Wrestling	57	21.543	3.190	15.291	27.795		
	Ping-pong	25	22.360	3.134	16.217	28.503		
	Badminton	20	22.700	4.001	14.858	30.542		
	Swimming	31	22.548	3.452	15.782	29.314		
Knowledge	Male	218	88.403	7.757	73.199	103.607	0.992	0.001
	Female	102	88.410	5.113	78.389	98.431		
Attitude	Male	218	28.779	5.812	17.387	40.171	0.533	0.079
	Female	102	28.382	4.005	20.532	36.232		
Performance	Male	218	26.133	3.490	19.293	32.973	0.548	0.075
	Female	102	26.382	3.374	19.769	32.995		

*CI* Confidence Interval

^*^Cohen’s d effect size

In ANOVA analysis conducted in this study, a common interpretation of effect sizes is that partial eta squared (η^2^) values of 0.01, 0.06, and 0.14 represent small, medium, and large effects, respectively. These effect size interpretations provide valuable insights into the magnitude of the relationships between variables, allowing for a better understanding of the impact of different factors on the outcomes under investigation.

The results of one sample t-test in Table 4, comparing the nutritional knowledge of athletes’s knowledge with the optimal level, indicate that the mean nutritional value of athletes’ knowledge is 79.594 ± 7.015, which is lower than the optimal level (84); therefore, it is worth mentioning that the average nutritional knowledge of athletes is weak (t = -11.236) (*p* > 0.05). The results of one sample t-test in Table 4 also indicate that the mean nutritional value of athletes’ attitude is 23.347 ± 5.300, which is lower than the optimal level (26); therefore, the mean nutritional attitude of athletes is weak (*p* < 0.05) (t = -8.954). The study’s optimal levels were tailored and customized to align with the characteristics and behaviors of the athlete population, following consultations with academic experts specialized in the field.

**Table 4:** Comparison of athletes’ nutritional knowledge, attitude, and performance with the optimal level

	The optimal level	t-value	Degrees of freedom	*P*-value	Mean differences	95% confidence interval for mean differences		Effect size^*^
						Lower bound	Upper bound	
Knowledge	84	0.236	319	0.001	-4.406	-3.634	-5.177	0.628
Attitude	26	8.954	319	0.001	-20.653	-2.070	-3.236	1
Performance	24	-11.471	319	0.001	-2.212	-1.833	-2.592	1.025

^*^Cohen’s d effect size

The one sample t-test results in Table 4 also highlight that the mean nutritional value of athletes’ performance is 21.788 ± 3.450, which is lower than the optimal level (24); hence, the mean nutritional performance of athletes is moderate (*p* < 0.05) (t = -11.471). For knowledge, attitude, and performance, the calculated effect sizes were 0.628, 1, and 1.025, respectively. An interpretation of effect sizes by Cohen [34] is that values of 0.01, 0.06, and 0.14 represent small, medium, and large effects, respectively.

### Correlation analysis

The results of the correlation analysis for the variables knowledge, attitude, and performance are presented in Table 5. The correlation analysis revealed several significant associations between the study variables. First, knowledge showed a moderate positive correlation with attitude (*r* = 0.302, *p* < 0.001), indicating that participants with higher levels of knowledge tend to have more positive attitudes. Second, the correlation between knowledge and performance was found to be statistically significant (*r* = 0.113, *p* < 0.05), suggesting a positive relationship between knowledge and performance. Lastly, attitude and performance were also positively correlated (*r* = 0.260, *p* < 0.001), indicating that individuals with more positive attitudes tended to perform better. It is important to note that correlation does not imply causation, and further investigations would be needed to establish the direction of these relationships. Nevertheless, the significant associations observed in this study provide valuable insights into the interplay between knowledge, attitude, and performance.

**Table 5:** The relationship between athletes’ nutritional KAP components

Variables	Knowledge	Attitude	Performance
Knowledge			
Pearson’s correlation	1		
*P*-value			
Attitude			
Pearson’s correlation	0.302**	1	
*P*-value	*p* < 0.001		
Performance			
Pearson’s correlation	0.113*	0.260**	1
*P*-value	0.044	*p* < 0.001	

^*^Means the correlation is significant at the 0.05 level (2-tailed), and **means the correlation is significant at the 0.01 level (2-tailed)

## Discussion

### Nutritional knowledge of professional athletes

The study found that the nutritional knowledge of professional athletes in the Iranian population was suboptimal. This indicates that, on average, athletes have only moderate nutritional knowledge. The wide range of scores suggests that there is considerable variation in knowledge levels among athletes.

Various studies highlight disparities in nutritional knowledge among different athlete populations. Vázquez-Espino et al. [24] found low median scores among professional soccer players in Spain (25.1), resembling high school students (19.5) and university Philosophy students (29.0). Contrarily, sports technical teams (58.5) and final year Human Nutrition and Dietetics students (74.6) scored significantly higher. Ahmadi et al. [35] observed a mean nutrition knowledge of 66% of high school athletes in Tehran. Trabucco et al. [36] reported good nutritional knowledge in Italian medical school student-athletes (66.7%) and slightly lower in Serbian students (63%). Nazni and Vimala [37] found varying levels among Indian athletes: volleyball players (42% good knowledge), weightlifters (43% satisfactory), and runners (29% very good). Conversely, Sangeetha and Ramaswamy [38] noted a lack of proper nutrition knowledge among college athletes in India. Folasire et al. [32] reported that 58.2% of Nigerian undergraduate athletes have good nutritional knowledge. Shakeel et al. [39] found 75.2% of Pakistani university sports students had good knowledge of healthy dietary practices. Ali et al. [40] highlighted disparities between male (fair knowledge, 57%) and female (poor knowledge, 49%) university student-athletes in Pakistan. Serhan et al. [41] noted inadequate nutrition knowledge (mean < 75%) among university athletes in Lebanon. Bakhtiar et al. [23] revealed 57.3% satisfactory nutrition knowledge among adolescent trainee athletes in Bangladesh. Sunuwar et al. [42] found over half Nepalese Taekwondo players with poor nutrition knowledge. Kathure et al. [43] indicated that most elite athletes in Kenya had adequate nutrition knowledge (above 50%). Aishwarya [44] reported a mean score of 51.9% in nutritional knowledge among physical education students in India. Bio Nigan et al. [45] observed good hydration performance and high knowledge levels among handball player students compared to non-athletes. Dunnigan et al. [46] highlighted low nutritional knowledge among Clemson University athletes (mean score of 11.59 ± 3.14 or 46.3%).

The trend of suboptimal nutritional knowledge among athletes is concerning, as proper nutrition is crucial for optimizing athletic performance. Athletes need to be well-informed about the role of various nutrients, dietary requirements, and strategies to meet their specific performance goals. The variation in scores may highlight the need for targeted nutrition education interventions to address the knowledge gaps among athletes.

### Nutritional attitude among professional athletes

The current study revealed that professional athletes displayed weak nutritional attitudes. This indicates that, on average, athletes had a less favorable attitude towards nutrition. Despite the results of the current study displaying weak nutritional attitude, many other studies showed good of fair levels of attitude in athletic populations.

Several studies have evaluated athletes’ attitudes toward nutrition. Ahmadi et al. [35] found that high school athletes in Iran had a nutrition attitude mean of 63.4%. Trabucco et al. [36] observed positive attitudes and good nutritional knowledge among students practicing sports in Italy and Serbia. Shakeel et al. [39] noted that 64.4% of participants had a positive attitude toward making proper food choices. Bakhtiar et al. [23] found a 57% positive attitude among Bangladeshi athletes. Aishwarya [44] reported excellent attitudes (41%) among Indian physical education students regarding food and nutrition. On the other hand, Dunnigan et al. [46] showed low nutritional attitudes among athletes, while Azizi et al. [47] recorded varied mean nutritional attitudes among male and female athletes. Bio Nigan et al. [45] found no difference in attitude between athlete and non-athlete groups.

Despite no significant difference between genders in the current study, both male and female athletes exhibited weak nutritional attitudes. Nutritional attitudes can significantly impact dietary practices and, consequently, athletic performance. The lack of positive attitudes toward nutrition highlights the importance of promoting a positive nutritional mindset among athletes. The trend of weak nutritional attitudes among athletes suggests that they may not prioritize nutrition as a key factor in their athletic performance. Positive attitudes towards nutrition are essential for promoting healthy dietary practices and adherence to proper nutrition guidelines. Improving athletes’ attitudes towards nutrition may lead to better dietary choices and, ultimately, enhanced performance.

### Nutritional practice of professional athletes

The present study found that the mean nutritional performance score of professional athletes indicated moderate dietary practices. The moderate nutritional performance score indicates that athletes’ actual dietary practices are relatively better than their knowledge and attitudes. However, there is room for improvement in dietary behaviors. Education on implementing sound nutritional knowledge into daily practices is crucial for optimizing. The following studies demonstrate moderate and good levels of athletic nutritional practice.

Monte Calbo et al.  [48] examined college athletes’ nutritional knowledge and performance in Filipino population. Their results demonstrated that their nutritional performance was moderate, and there was no significant relationship between age and gender. They also showed a significant relationship between nutritional knowledge and performance; thus, athletes with higher nutritional knowledge had better nutritional performance. The results of the study by Folasire et al. [32] demostrate good nutrition practices (62.7%) in Nigerian athletes. In the study by Bakhtiar et al. [23] the participants displayed good nutrition practices (57.69%). Although the study by Bio Nigan et al. [45] monitored hydration and not other aspects of dietary practices, it still showcases how high knowledge can contribute to elevated levels of athletic performance. The study by Ahmadi et al. [35] also showed mean values for nutrition practice was 48.71%.

However, in the study by Shakeel et al. [39], the actual practices of the participants were poor and did not align with their good knowledge and attitude level; only about 57.4% of the students implemented their knowledge in their practices, suggesting a gap between knowledge and behavior. Sunuwar et al. [42] also indicated a poor level of nutrition practice (55.3%) in Taekwondo athletes in Nepal.

Although the trend toward moderate nutritional performance implies that while athletes may have a relatively better grasp of putting their nutritional knowledge into practice, there is still room for improvement. Implementing sound nutritional practices can consistently have a significant impact on athletes’ overall health and performance. By enhancing their dietary practices, athletes can potentially achieve better athletic outcomes.

### Relationship between nutritional KAP components

The findings of the present study revealed an association between these key variables of KAP, reinforcing the interconnectedness of these components. The correlation analysis demonstrated a positive and statistically significant relationship among the different components of KAP (*p* < 0.05). Knowledge and attitude were positively correlated, with a moderate correlation coefficient of *r* = 0.302. This indicates that athletes who possessed higher levels of nutritional knowledge tended to have more positive attitudes towards nutrition, and vice versa. This highlights the fact that a better understanding of nutrition may foster a more positive attitude towards making healthier dietary choices.

Furthermore, the correlation analysis also established a positive but weak relationship between knowledge and performance (*r* = 0.113). This result suggests that while there is some degree of association between nutritional knowledge and actual dietary performance, the relationship is not as strong as the knowledge-attitude association. Nevertheless, it still indicates that athletes with greater nutritional knowledge are more likely to exhibit improved dietary practices, contributing to better overall performance in their respective sports.

Also, a positive and moderate correlation was observed between attitude and performance (*r* = 0.260). This suggests that athletes with more positive attitudes towards nutrition are likely to demonstrate better nutritional practices, resulting in enhanced performance outcomes. The positive association between attitude and performance supports the significance of mental and psychological factors in influencing athletes’ dietary choices and, subsequently, their athletic achievements.

### Potential reasons for low nutritional KAP scores

Nutritional knowledge and behavior are not the only factor affecting the nutritional performance of individuals and can be influenced by several factors. The potential impact of the education system, media, and social factors can also contribute to low levels of athletes’ nutritional KAP. Misleading information from unreliable sources may hinder athletes’ ability to make informed dietary decisions; therefore, implementing nutrition education programs and promoting evidence-based nutritional information can help athletes distinguish accurate advice from misinformation. Other factors including the person’s physiological needs, mental self-image, access to food, social media influences, food preferences, and the performance of friends and relatives also affect nutritional performance [49].

### Limitations of the study

Despite some limitations in the study, such as incomplete coverage of all sports clubs and imbalances in age and academic level groups, the research provides valuable insights into athletes’ nutritional KAP in Qazvin province, Iran. Future studies on a larger scale can further explore athletes’ nutritional knowledge and mindset and evaluate other dietary factors that impact athletic performance, including food availability, socio-economic variables, and micronutrients.

### Recommendations and future directions

Previous studies have also shown the positive impact of nutritional knowledge on dietary practices and performance. However, this study observed lower-than-optimal nutritional KAP among athletes, indicating a need for further improvement and education.

#### Nutrition education and support

The study suggests practical dietary recommendations to improve athletes’ nutritional knowledge, such as obtaining information from reliable academic sources and not relying on myths or word-of-mouth. Incorporating nutrition education into the training programs of professional sports clubs is essential. Hiring certified informative nutritionists in sports clubs could also help enhance developing personalized dietary plans for athletes, considering their specific nutritional needs and performance goals, subsequently elevating athletes’ nutrition knowledge and performance.

#### Further research

Future studies should explore nutritional KAP on a larger scale, including national and international populations of athletes as this issue is not unique to Iran and it underscores the need for global efforts to improve athletes’ nutritional knowledge and practices. Additionally, evaluating the impact of micronutrients on athletic performance could provide valuable insights into optimizing dietary practices.

## Conclusions

In summary, this study sheds light on the nutritional KAP of professional athletes in Qazvin, Iran, and emphasizes the importance of improving their nutritional knowledge and attitude to enhance performance. The findings of this study indicate that while athletes recognize the importance of nutrition and its impact on athletic health and performance, their overall nutritional KAP remains at a moderate or weak level, falling short of the optimal standard. Accurate nutrition education and support from certified nutritionists in sports clubs could play a crucial role in achieving this goal. With a better understanding of nutrition, athletes can make informed food choices and optimize their performance, ultimately contributing to their overall health and success in their respective sports.

### Supplementary Information

**Additional file 1.** Preliminary Interview Questions.

## Data Availability

The data presented in this study are available on request from the corresponding author. The data are not publicly available due to ethical concerns.
