# Supplementary material for: Nutritional knowledge, attitude, and practice of professional athletes in an Iranian population (a cross-sectional study)
Source: BMC Sports Sci Med Rehabil. 2023 Dec 4;15:164. doi: 10.1186/s13102-023-00776-3 (PMC10696817; doi:10.1186/s13102-023-00776-3)
Supplement: Supplementary file 1 — Additional file 1. Preliminary Interview Questions. [file 13102_2023_776_MOESM1_ESM.pdf]

Preliminary Interview Questions

This supplementary document comprises the preliminary interview questions, custom-developed for this study to comprehensively capture essential demographic information and contextual details relevant to the nutritional practices of professional athletes alongside the main demographic questionnaire.

The interview aimed to inform about the study procedure and gather specific details to help decide whether the participant meets the inclusion criteria. The design of these questions was meticulously crafted to align with the study's objectives, ensuring a nuanced understanding of the factors influencing athletes' nutritional knowledge, attitudes, and practices. While the questions were newly formulated for this research, they were informed by the broader context of nutritional studies and tailored to suit the unique requirements of our investigation. This approach allowed us to collect rich and relevant data to contribute meaningfully to the sports nutrition field.

By introducing participants to these inquiries, we aimed to establish a robust foundation for subsequent data analysis and interpretation. The design of the interview questions aligns with the study's overarching goals, fostering a detailed exploration of factors influencing nutritional knowledge, attitudes, and practices among professional athletes in Qazvin, Iran.

1. **General Information:**
    - What is your age?
    - What is your gender?
    - What is your sports field?
    - What is your marital status?
    - What is your education level?
    - What is your current career or profession outside of sports?
2. **Training and Exercise Routine:**
    - How many days per week do you engage in structured exercise?
    - What is the average duration of your training sessions?
    - Could you describe your typical exercise routine?
    - Have you maintained this exercise frequency and duration for at least three years?
3. **Competition Experience:**
    - How many years have you been engaged in professional sports?
    - Have you competed in national or provincial matches?

- Could you provide details about your competition experience?

4. **Nutritional Practices and Weight Changes:**
   - Do you follow a specific diet or nutritional plan?
   - Are you currently taking any supplements?
   - Do you have any dietary restrictions or preferences?
   - Have you experienced any significant weight gain or loss in the last four months?
5. **Health and Medical History:**
   - Do you have any medical history that may affect your participation in this study?
   - Do you have any pre-existing medical conditions?
   - Do you take any supplements?
   - Do you follow a specific diet?
   - Are you currently on any medication?
   - Do you have any gastrointestinal tract disorders or diseases? Have you ever experienced any?
6. **Lifestyle Factors:**
   - What is your typical daily routine?
   - How do you manage stress in your life?
   - Are there any specific challenges you face in maintaining a balanced lifestyle?

We sincerely appreciate all participants for their invaluable cooperation throughout the preliminary interviews. Your willingness to share essential information significantly contributes to the depth and reliability of this study. Thank you for your time and collaboration.
